# Comorbidities and Health-Related Quality of Life in Subjects with Spine Osteoarthritis at 50 Years of Age or Older: Data from the Korea National Health and Nutrition Examination Survey

**DOI:** 10.3390/medicina58010126

**Published:** 2022-01-14

**Authors:** Seong-Kyu Kim, Jung-Yoon Choe

**Affiliations:** Division of Rheumatology, Department of Internal Medicine, Catholic University of Daegu School of Medicine, Daegu 42472, Korea; jychoe@cu.ac.kr

**Keywords:** osteoarthritis, spine, comorbidity, quality of life

## Abstract

*Background and Objective:* This study assessed comorbidities and health-related quality of life (HRQOL) in subjects with lumbar spine osteoarthritis (OA) in the Korean population. *Materials and Methods:* We analyzed 3256 subjects who were 50 years or older and underwent plain radiography of the lumbar spine as part of the Korea National Health and Nutrition Examination Survey (KNHANES) 2012. Radiographic assessment was based on Kellgren–Lawrence (K-L) grade ranging from 0 to 2, with K-L grade 2 defined as lumbar spine OA. HRQOL was assessed by EuroQol-5 dimensions (EQ-5D), which include the EQ-5D index and visual analogue scale (EQ-VAS) measurements. *Results:* Comorbidities such as hypertension, myocardial infarction, angina, cerebral infarction, and diabetes mellitus were more frequent in spine OA than in controls, while dyslipidemia was less common. Subjects with spine OA had higher mean number of comorbid conditions than controls (1.40 (SE 0.05) vs. 1.20 (SE 0.03), *p* = 0.001). Subjects with spine OA had much lower EQ-5D index than controls (*p* < 0.001) but not lower EQ-VAS score. Multivariate binary logistic analysis showed that hypertension and colon cancer were associated with spine OA compared to controls (OR 1.219, 95% CI 1.020–1.456, *p* = 0.030 and OR 0.200, 95% CI 0.079–0.505, *p* = 0.001, respectively) after adjustment for confounding factors. Lower EQ-5D index was related to spine OA (95% CI 0.256, 95% CI 0.110–0.595, *p* = 0.002) but not EQ-VAS score. *Conclusion*: In this study, we found that comorbidities such as hypertension and colon cancer as well as lower HRQOL were associated with spine OA.

## 1. Introduction

Osteoarthritis (OA) of the spine is a common form of arthritis affecting intervertebral discs, vertebral bodies, and zygapophyseal joints in the spinal structure [1,2]. OA can lead to significant morbidity and functional disability over time. Pathologic abnormalities in spine OA include degenerative change of intervertebral discs, deforming spondylosis with vertebral osteophyte formation, and arthritis at the zygapophyseal joints [2]. Lumbar spine OA is considered prevalent, estimated to range from 20% to 85% in the elderly population [3,4,5], due mainly to characteristics of the study population and definition of disease. Low back pain, a typical clinical feature in spine OA, is a common condition motivating visits to healthcare centers and is the second most common cause of disability among the adult US population, with estimates of about 80% affected at sometime during life [6]. A significant proportion of subjects with spine OA is more likely to experience debilitating conditions and poor quality of life.

Recently, the clinical significance of comorbidities in OA has been recognized due to potential effects of comorbid conditions on clinical practices and outcomes, including optimal treatment strategy, expectation of prognosis, health-related quality of life (HRQOL), and health care expenditures [7,8,9,10]. Increases in the prevalence of comorbidities in OA have been observed, with estimates ranging from approximately 50% to 60% of OA patients with at least one comorbidity. Recognition of comorbid conditions in patients with OA might be helpful to reduce pain, physical disability, and disease burden and subsequently improve quality of life [9,10]. Chronic spinal pain is associated with increased probability of comorbidities or decreased HRQOL [11,12]. Lumbar lordosis was found to be a risk factor for reduced physical quality of life in a Japanese study [13]. However, there are insufficient data about comorbid conditions and HRQOL in spine OA. Thus, the objective of this study is to determine the relationships of radiographic spine OA with comorbidities and quality of life in the Korean population.

## 2. Subjects and Methods

### 2.1. Study Population

The Korea National Health and Nutrition Examination Survey (KNHANES) is a national cross-sectional survey conducted by the Korea Centers for Disease Control and Prevention to assess population-based health and nutrition in the non-institutionalized Korean population. The KNHANES is conducted by special research teams using individual interviews. Survey participants were selected using a stratified multi-stage cluster probability sampling model to extract representative data about general health and nutritional status in the Korean population after application of the power of sample weight.

For this study, we used data from KNHANES 2012 that are available on the official KNHANES web site (https://knhanes.kdca.go.kr/knhanes/sub03/sub03_02_05.do). Among a total of 8518 subjects who participated in KNHANES 2012, we initially selected 3444 subjects 50 years of age or older who had undergone radiographic assessments of the lumbar spine and excluded 188 subjects without radiographic results. Therefore, data from 3256 subjects were collected and analyzed. Written informed consent was obtained from all participants at the time of enrollment.

### 2.2. Clinical Information

Information for demographic and socioeconomic variables were obtained from interviews with each individual using a well-designed questionnaire for KNHANES 2012. These data included age, sex, body mass index (BMI, kg/m^2^), marital status (unmarried vs. married), education level (elementary school or less, middle school, high school, and college or higher), income status (low, mid-low, mid-high, and high), alcohol consumption (non-alcoholic vs. alcoholic), and smoking status (never smoker, ex-smoker, and current smoker). BMI is calculated as weight in kilograms divided by height in meters squared. Income level was determined by defining quartiles based on the average monthly income of the study participants.

The questionnaire included questions about comorbid disease, and we confirmed physician diagnoses for all included data. Non-malignant comorbidities included hypertension, myocardial infarction/angina, cerebral infarction, dyslipidemia, pulmonary tuberculosis, bronchial asthma, diabetes mellitus, thyroid disease, depression, atopic dermatitis, chronic renal failure, hepatitis B, hepatitis C, and liver cirrhosis. In addition, malignancies considered comorbidities including gastric cancer, liver cancer, colon cancer, breast cancer, cervical cancer, lung cancer, and thyroid cancer were identified.

### 2.3. Assessment of Health-Related Quality of Life

Generic health-related quality of life was evaluated using the EuroQOL-5 dimension-3 level (EQ-5D-3L) questionnaire, the EQ-5D index, and the EuroQOL-visual analogue scale (EQ-VAS). The validity and reliability of the EQ-5D were approved as a simple instrument for measuring HRQOL in the Korean population [14]. The EQ-5D is an acceptable and valid measure for assessment of HRQOL in Korean patients with OA [15].

### 2.4. Radiographic Assessment

Among the total study population, radiographic examinations of the lumbar spine were performed on subjects 50 years of age or older. The radiographic severity of the lumbar spine was evaluated based on Kellgren–Lawrence (K-L) criteria [16]. The K-L grade was classified originally as grade 0 to grade 4 for the knee joint but was modified to grade 0 to grade 2 for the lumbar spine. Radiographic grades for the lumbar spine were determined as follows: K-L grade 0 for normal, K-L grade 1 for suspicious (osteophytes), and K-L grade 2 for abnormal (intervertebral disc space narrowing, bone sclerosis, or large osteophyte) [17]. Radiographic K-L grade 2 was diagnosed as spine OA, and K-L grades 0 and 1 were considered no spine OA.

### 2.5. Statistical Analysis

Qualitative variables were described as non-weighted number of cases (weighted %), and quantitative variables were described as mean (standard error, SE). Comparisons of qualitative variables were assessed by a composite sample chi-square analysis, whereas a two-sample *t*-test or Fisher’s exact test was applied to compare quantitative variables.

To analyze the effect of spine OA on the comorbidities and HRQOL, multivariate logistic regression models were performed with malignant or non-malignant comorbidities and HRQOL as dependent variables and spine OA as an independent variable. Associations between variables and spine OA were evaluated by composite sample binary logistic regression analysis after adjustment for confounding variables including age and gender in model 1 and age, gender, marital status, education level, income level, alcohol consumption, and smoking in model 2. The results are presented as 95% confidence intervals (CIs) and odds ratios (ORs). Statistical analyses were performed with IBM SPSS Statistics 19.0 (IBM Corp., Armonk, NY, USA). *p* values less than 0.05 were considered statistically significant.

## 3. Results

### 3.1. Baseline Characteristics of the Study Population

The baseline characteristics of a total of 3256 subjects are described in Table 1. The mean age of the study population was 62.3 (SE 0.2) years, and 1858 subjects (53.8%) were female. The mean BMI was 24.0 kg/m^2^ (SE 0.1). Data for other characteristics including marital status, education level, income level, alcohol consumption, and smoking are included in Table 1.

We identified frequencies of non-malignant comorbid conditions such as hypertension, myocardial infarction/angina, cerebral infarction, dyslipidemia, bronchial asthma, pulmonary tuberculosis, diabetes mellitus, thyroid disease, depression, atopic dermatitis, chronic renal failure, viral hepatitis, and liver cirrhosis. The frequencies of malignant comorbidities such as gastric cancer, liver cancer, colon cancer, breast cancer, cervical cancer, lung cancer, and thyroid cancer also were identified. Among comorbidities, hypertension (38.2%) was the most common, followed by depression (18.1%) and dyslipidemia (17.3%). The frequencies of malignant comorbidities were estimated to range from 0.1% to 1.1%. In assessment of HRQOL, EQ-VAS score was 85.8 (SE 2.9), and EQ-5D index was 0.90 (SE 0.00).

### 3.2. Comparison of Characteristics between Spine OA and Controls

Among a total of 3256 subjects enrolled in this study, 1141 had spine OA (32.3%, SE 1.1), and 2115 were control subjects (without spine OA) (67.7%, SE 1.1) (Table 1). Subjects with spine OA were older (*p* < 0.001) and more likely to be female (*p* = 0.001). There was no difference in BMI between groups. Other variables including marital status, education level, income level, alcohol consumption, and smoking status were significantly different between the two groups.

**Figure 1:**
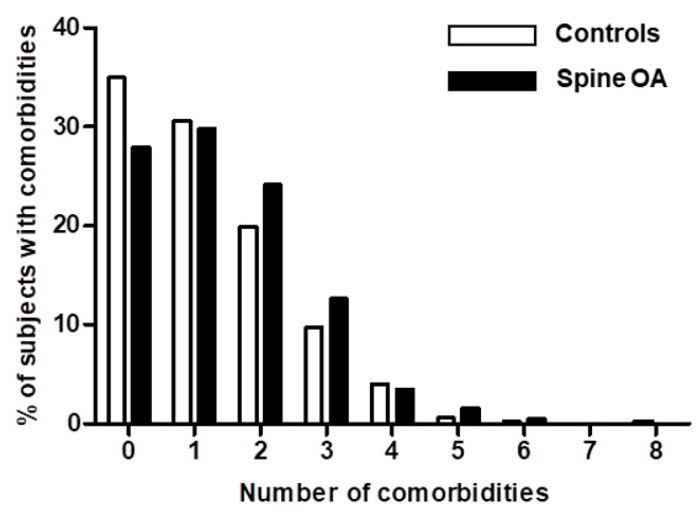
Distribution of proportion of controls and subjects with spine OA according to the number of comorbidities. Abbreviation: OA, osteoarthritis.

Comparing the frequencies of comorbidities, subjects with spine OA had higher frequencies of hypertension, myocardial infarction/angina, cerebral infarction, and diabetes mellitus than controls (*p* < 0.001, *p* = 0.022, *p* = 0.016, and *p* = 0.029, respectively), whereas dyslipidemia was more common in controls (*p* = 0.005). In contrast, there were no differences of malignancies between controls and spine OA. The mean number of comorbid conditions in spine OA was 1.40 (SE 0.05), and that in controls was 1.20 (SE 0.03), a significant difference (*p* = 0.001). In addition, the control group had a significantly higher proportion of participants without comorbidities than the spine OA group (35.0% vs. 28.0%) (Figure 1). As the number of comorbidities increased, the proportion of spine OA subjects tended to increase compared to controls.

**Figure 2:**
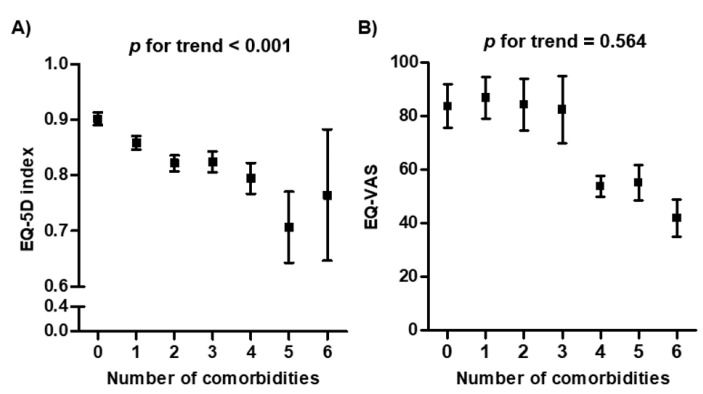
Distribution of health-related quality of life according to the number of comorbidities in subjects with spine OA (**A**) EQ-5D index according to comorbidities. (**B**) EQ-VAS according to comorbidities. Abbreviation: EQ-VAS score, EuroQOL visual analogue scale scores; EQ-5D index, EuroQOL-5dimension index.

In comparison of HRQOL, spine OA was associated with significantly lower EQ-5D index than were controls (*p* < 0.001) (Table 1). However, there was no difference in EQ-VAS score between groups (*p* = 0.414). As the number of comorbidities increased in spine OA, the EQ-5D index gradually and significantly decreased (*p* for trend < 0.001) (Figure 2A). However, the relationship between number of comorbidities and EQ-VAS was not significant (Figure 2B).

Data were described as mean and standard error.

### 3.3. Relationships between Spine OA and Comorbid Conditions

In the multivariate binary logistic regression analysis in the comparison of spine OA and controls, there was a decreased risk of pulmonary tuberculosis in spine OA in model 1 (OR 0.685, 95% CI 0.495–0.947, *p* = 0.022) (Table 2). In contrast, spine OA was not associated with the risk of other non-malignant comorbid conditions. In model 2, spine OA was significantly associated with an increased risk of hypertension (OR 1.219, 95% CI 1.020–1.456, *p* = 0.030), but it was not linked with those of other comorbidities.

In the logistic regression analysis with malignant comorbidities as the dependent variables in the comparison of spine OA and controls, model 1 revealed that spine OA showed lower risk of colon cancer (OR 0.185, 95% CI 0.077–0.443, *p* < 0.001) compared to controls (Table 3). Similarly, spine OA had a negative association with the risk of colon cancer (OR 0.200, 95% CI 0.079–0.505, *p* = 0.001) in model 2.

In the comparison of spine OA and controls, there was an increased risk of lower EQ-5D index score in spine OA in model 1 (OR 0.272, 95% CI 0.121–0.615, *p* = 0.002), but it was not related to EQ-VAS score (*p* = 0.478) (Table 4). Consistently, model 2 confirmed that spine OA was related with lower EQ-5D index (OR 0.256, 95% CI 0.110–0.595, *p* = 0.002), but it was not related to EQ-VAS score (*p* = 0.470).

## 4. Discussion

Although many studies have been conducted on the relationships between OA and comorbidities, there remain debates about the nature of the relationship. Moreover, previous studies have focused on patients with knee or hip OA, and there are insufficient studies on the roles of comorbidities and HRQOL in spine OA. In this study, we investigated comorbidities and quality of life in subjects over 50 years of age with radiographic spine OA using data from a national population-based health and nutrition survey. The main finding of this study was that comorbid diseases such as hypertension and colon cancer were associated with radiographic spine OA as defined using the K-L grade scale. In addition, we found that subjects with spine OA are vulnerable to poor quality of life, as shown by lower EQ-5D index.

Growing evidence suggests a close relationship between hypertension and OA. A meta-analysis of eight cohort or cross-sectional studies with 9765 subjects found significant association between hypertension and radiographic or symptomatic knee OA [18]. In an analysis of data from a 3-year follow-up of the ROAD study, hypertension was significantly responsible for development and progression of knee OA (OR 2.74, *p* = 0.008 and OR 1.54, *p* = 0.012, respectively) [19]. A nationwide study of registered subjects who underwent primary total hip or knee replacements found that hypertension was significantly associated with early revision after total knee joint replacement surgery, suggesting that hypertension might contribute to poor clinical outcomes after knee surgery [20]. Consistent with these findings, we also found a close association between spine OA and hypertension, although studies of this relationship have not been conducted in individuals with lumbar spine OA. However, the relationship between hypertension and risk of OA remains controversial. Low systolic blood pressure was significantly associated with OA at the knee, hand, and hip joint in an analysis of data from 384,838 unrelated participants in the UK Biobank study [21]. The precise mechanism underlying the relationship between hypertension and OA has not been determined. Clinically, metabolic syndrome and its components have been regarded as independent risk factors for knee or hip OA [22]. Considering that hypertension is a metabolic syndrome component, it might be clinically linked to spine OA. In terms of vascular pathogenic mechanisms, OA is a joint disease characterized by impaired perfusion to subchondral bone and ischemia in local joint tissues, which results in development of hypoxia and lack of nutritional supply within articular structures [23]. In addition to disruption of the local vascular environment, systemic vascular abnormalities such as hypertension could play an important role in the pathogenesis of OA. In an animal study, hypertension was found to be potentially responsible for disturbances of balance in both arterial and venous blood flow [24]. Furthermore, increased interosseous pressure and disturbance of perfusion by hypertension led to dysregulation of bone remodeling in response to mechanical stress [25]. Based on this evidence, hypertension might contribute to the development of OA through derangement of local vascular homeostasis in joint structure components of the spine.

It has been well established that systemic inflammation plays a crucial role in the pathogenesis of atherosclerosis and development of cardiovascular diseases [26]. Recently, evidence has been presented that OA is a low-grade inflammatory disease [27]. Evidence supporting the association of OA with cardiovascular disease has accumulated. A meta-analysis of the potential association between OA and cardiovascular disease using 15 observational studies demonstrated that the risk of cardiovascular disease increased by 24% in patients with OA compared to the general population (relative risk 1.24, *p* < 0.001) [28]. In a prospective study from the Progetto Veneto Anziano Study Cohort, subjects with OA were more likely to develop new onset cardiovascular diseases than were those without OA, and the presence of OA contributed to increased risk of cardiovascular disease [29]. Based on these findings, OA is a significant risk factor or predictor of cardiovascular disease. In contrast, OA was not associated with coronary heart disease risk factors or with its 8-year incidence in a sample of elderly Japanese American males who participated in the Honolulu Heart Program’s fourth examination from 1991 to 1993 [30]. Consistent with the result of that observational study in Japanese American subjects, our study did not detect relationships between cardiovascular diseases such as ischemic heart disease or cerebral infarction and OA.

There is lack of epidemiologic data about malignancy and OA. In the nationwide Danish Hospital Discharge Registry, the risk of cancer did not increase among subjects who received knee or hip joint replacements [31]. Radiographic knee OA with pain was not associated with malignancies including lung cancer, cervical cancer, breast cancer, colon cancer, and stomach cancer compared to non-knee OA in KNHANES data [32]. In contrast, increased cancer risk was evident in the liver, pancreas, breast, and bladder among subjects with knee OA aged 40 years or older in the Information System for Research in Primary Care (SIDIAP) database [33]. Ward et al. found that patients with knee or hip OA were at lower risk of colorectal and lung cancer compared to the general population in a retrospective cohort study [34]. Similar to prior results, we also observed lower risk of colon cancer in spine OA patients compared to controls. To explain the low risk of colon cancer in subjects with spine OA, consideration should be given to the use of medications such as nonsteroidal anti-inflammatory drugs (NSAIDs). Long-term use of non-selective NSAIDs has been linked to lower risk of gastrointestinal cancers [35]. Although the KNHANES data did not provide clinical information regarding the use of NSAIDs, we assumed that medications used to treat spine OA such as NSAIDs were related to lower risk of colon cancer.

OA is a disease that has a profound impact on quality of life because the disease is chronic and sometimes incurable and can lead to disability [36]. HRQOL assesses each subject’s functional status in daily life and subjective perceptions of well-being. To measure HRQOL in this study, we used EQ-5D. We found that subjects with spine OA had lower EQ-5D index compared to those without spine OA. Consistently, doctor-diagnosed knee OA was negatively associated with HRQOL assessed using the Knee Injury and Osteoarthritis Outcome Score-Quality of Life [37]. Jeong et al. also observed lower EQ-5D index in knee OA than in patients without knee OA and negative associations between HRQOL indexes and knee OA [32]. In a comparison between older adults with hip and/or knee OA and those without OA, subjects with OA of the lower extremities showed significant effects on multiple dimensions of the Study Short Form-36 item health status questionnaire (SF-36) [38]. Consistent with the findings of our study, Imagama et al. observed negative impact of increased comorbidities on physical QOL in patients with lumbar lordosis [13]. We also found no difference in EQ-VAS score between subjects with and without spine OA. In another study using KNHANES data, EQ-VAS was significantly lower in subjects with knee OA with pain than in subjects without knee OA [32]. However, subjects with knee OA but without knee pain had similar EQ-VQS as those without knee OA. As in our study, the reason underlying the similarity of EQ-VAS in spine OA and control group is hypothesized as follows. Osteophytes, one of the criteria used for classification of spine OA, is weakly related to low back pain, while disc space narrowing is moderately associated with back pain [39]. In addition to the vertebral body, the facet joints of the vertebrae can be associated with low back pain; in a recent study, facet joint OA confirmed by computed tomography was not associated with low back pain [40]. Additional research on the relationship between spine OA and low back pain is needed.

There are some limitations impairing the explanation of the relationship between comorbidity and spine OA. First, detailed information about OA-related medications was lacking from KNHANES 2012. Our main finding, that hypertension and colon cancer were associated with spine OA, might be attributable to the use of therapeutic drugs such as aspirin or NSAIDs. Second, our ability to identify other comorbidities present in other body organs and tissues in subjects with spine OA was limited because the types of comorbidities in this study were not well-defined. Third, we lacked a well-designed questionnaire about back pain, which would be necessary to explain why we observed no difference in EQ-VAS between spine OA patients and controls. It is difficult to confirm the relationship between back pain and EQ-VAS because the questionnaire did not include a clear definition of low back pain. Fourth, another limitation is the lack of information about patterns of physical activity and sedentary behavior that can affect spine OA.

In conclusion, we found that subjects with spine OA were more likely to have comorbid conditions than subjects without spine OA. Hypertension was positively associated with spine OA among non-malignant comorbidities, whereas spine OA was associated with lower colon cancer risk than were the controls. In addition, subjects with spine OA showed lower EQ-5D index than controls, suggesting that spine OA had a potent negative impact on HRQOL. By identifying comorbidities and lower HRQOL in subjects with spine OA, physicians should be concerned about and address comorbidities and quality of life when managing patients with spine OA.

## Figures and Tables

**Table 1:** Comparison of variables between controls and subjects with spine OA (n = 3.256).

Variables	Total (n = 3.256)	Controls (n = 2.115)	Spine OA (n = 1.141)	*p*-Value *
Age (years)	62.3 (0.2)	59.8 (0.3)	67.5 (0.4)	<0.001
Gender (female, n, %)	1858 (53.8)	1161 (50.7)	697 (60.1)	0.001
Body mass index (kg/m^2^)	24.0 (0.1)	24.0 (0.1)	23.9 (0.1)	0.988
Marital status (n, %) ^†^				0.001
Unmarried	695 (19.4)	33 (2.0)	3 (0.3)	
Married	2475 (80.6)	2078 (98.0)	1137 (99.7)	
Education (n, %) ^†^				<0.001
Elementary school or less	1501 (45.2)	808 (37.7)	693 (61.1)	
Middle school	539 (19.4)	379 (20.8)	160 (16.4)	
High school	753 (24.0)	579 (27.9)	174 (15.7)	
College or higher	380 (11.4)	302 (13.6)	78 (6.7)	
Income (n, %) ^†^				0.016
Low	806 (26.9)	513 (27.1)	293 (26.6)	
Mid-low	817 (26.5)	506 (25.5)	311 (28.6)	
Mid-high	808 (24.7)	526 (24.2)	282 (25.7)	
High	790 (21.9)	547 (23.2)	243 (19.1)	
Alcohol consumption (n, %) ^†^				<0.001
Non-alcoholic	695 (19.4)	401 (16.8)	294 (25.0)	
Alcoholic	2475 (80.6)	1668 (83.2)	807 (75.0)	
Smoking (n, %) ^†^				0.043
Never smoker	1842 (55.7)	1178 (53.5)	664 (60.3)	
Ex-smoker	788 (25.1)	519 (25.4)	268 (24.7)	
Current smoker	502 (19.2)	351 (21.1)	151 (15.0)	
Comorbidities ^†^				
Hypertension	1298 (38.2)	751 (33.2)	547 (48.8)	<0.001
Myocardial infarction/angina	170 (4.8)	97 (4.0)	73 (6.4)	0.022
Cerebral infarction	135 (3.9)	75 (3.2)	60 (5.5)	0.016
Dyslipidemia	623 (17.3)	436 (18.1)	187 (15.5)	0.005
Bronchial asthma	215 (6.7)	129 (6.2)	86 (7.7)	1.000
Pulmonary tuberculosis	281 (8.8)	196 (9.4)	85 (7.6)	0.091
Diabetes mellitus	485 (14.8)	295 (13.9)	190 (16.6)	0.029
Thyroid disease	167 (4.4)	107 (4.3)	60 (4.8)	0.756
Depression	584 (18.1)	377 (17.7)	207 (19.1)	0.722
Atopic dermatitis	82 (2.5)	56 (2.3)	26 (2.9)	0.550
Chronic renal failure	22 (0.6)	15 (0.6)	7 (0.5)	0.767
Hepatitis B	55 (1.7)	39 (2.0)	16 (1.2)	0.369
Hepatitis C	9 (0.2)	8 (0.2)	1 (0.2)	0.135
Liver cirrhosis	16 (0.4)	11 (0.5)	5 (0.2)	0.764
Gastric cancer	38 (1.1)	25 (1.0)	13 (1.2)	0.938
Liver cancer	4 (0.1)	3 (0.1)	1 (0.1)	0.566
Colon cancer	4 (0.1)	27 (1.3)	7 (0.5)	0.080
Breast cancer	34 (1.1)	16 (0.6)	5 (0.3)	0.288
Cervical cancer	21 (0.5)	13 (0.5)	6 (0.5)	0.767
Lung cancer	19 (0.5)	2 (0.1)	2 (0.1)	0.434
Thyroid cancer	4 (0.1)	18 (0.8)	4 (0.4)	0.100
EQ-VAS score	85.8 (2.9)	82.6 (3.2)	92.3 (6.3)	0.414
EQ-5D index	0.90 (0.00)	0.92 (0.00)	0.85 (0.01)	<0.001

Data were described as non-weighted number of cases (weighted %) for qualitative variables or mean (standard error, SE) for quantitative variables. Abbreviation: OA, osteoarthritis; EQ-VAS score, EuroQOL visual analogue scale scores; EQ-5D index, EuroQOL-5dimension index. * *p* values were obtained by two sample *t*-test or chi-square test or Fisher’s exact test. ^†^ Missing data were excluded from the analyses: for marriage, n = 5; for education, n = 83; for income, n = 35; for alcohol consumption, n = 86; for smoking, n = 124; comorbidities, n = 79.

**Table 2:** Multivariate-adjusted ORs (95% CIs) for non-malignant comorbidities in spine OA compared to controls.

	Model 1			Model 2		
Variables	OR	95% CI	*p* Value	OR	95% CI	*p*-Value
Hypertension	1.188	0.990–1.426	0.064	1.219	1.020–1.456	0.030
Myocardial infarction/angina	1.116	0.760–1.640	0.574	1.088	0.725–1.635	0.681
Cerebral infarction	1.083	0.657–1.787	0.753	0.068	0.635–1.798	0.802
Dyslipidemia	0.795	0.614–1.029	0.081	0.826	0.632–1.079	0.160
Bronchial asthma	0.940	0.646–1.367	0.744	0.937	0.640–1.373	0.738
Pulmonary tuberculosis	0.685	0.495–0.947	0.022	0.743	0.531–1.039	0.082
Diabetes mellitus	0.961	0.728–1.268	0.775	0.978	0.735–1.303	0.880
Thyroid disease	1.090	0.733–1.620	0.669	1.066	0.717–1.585	0.752
Depression	1.147	0.882–1.492	0.303	1.119	0.850–1.474	0.420
Atopic dermatitis	1.532	0.775–3.031	0.218	1.752	0.866–3.544	0.118
Chronic renal failure	0.700	0.238–2.060	0.515	0.659	0.234–1.859	0.429
Hepatitis B	0.865	0.478–1.565	0.630	0.874	0.483–1.581	0.654
Hepatitis C	0.571	0.043–7.594	0.669	0.664	0.054–8.148	0.748
Liver cirrhosis	0.564	0.203–1.571	0.272	0.531	0.183–1.547	0.244

Data were described as odds ratio (OR) and 95% confidence intervals (CIs). Abbreviation: OA, osteoarthritis. *p* values were obtained by the composite sample multivariate logistic regression analysis: Model 1, adjusted with age and gender; Model 2, adjusted with age, gender, marital status, education level, income level, alcohol consumption, and smoking.

**Table 3:** Multivariate-adjusted ORs (95% CIs) for malignant comorbidities in spine OA compared to controls.

	Model 1			Model 2		
Variables	OR	95% CI	*p* Value	OR	95% CI	*p* Value
Gastric cancer	0.829	0.421–1.632	0.586	0.763	0.373–1.562	0.457
Liver cancer	1.337	0.114–15.641	0.816	1.517	0.139–16.608	0.732
Colon cancer	0.185	0.077–0.443	<0.001	0.200	0.079–0.505	0.001
Breast cancer	0.314	0.093–1.067	0.063	0.333	0.101–1.094	0.070
Cervical cancer	0.674	0.138–3.281	0.623	0.463	0.065–3.307	0.441
Lung cancer	2.756	0.663–11.446	0.162	2.899	0.685–12.271	0.147
Thyroid cancer	0.641	0.232–1.774	0.390	0.639	0.227–1.799	0.394

Data were described as odds ratio (OR) and 95% confidence intervals (CIs). Abbreviation: OA, osteoarthritis. *p* values were obtained by the composite sample multivariate logistic regression analysis: Model 1, adjusted with age and gender; Model 2, adjusted with age, gender, marital status, education level, income level, alcohol consumption, and smoking.

**Table 4:** Multivariate-adjusted ORs (95% CIs) for health-related quality of life in spine OA compared to controls.

	Model 1			Model 2		
Variables	OR	95% CI	*p* Value	OR	95% CI	*p* Value
EQ-VAS score	1.000	0.999–1.001	0.478	1.000	0.999–1.001	0.470
EQ-5D index	0.272	0.121–0.615	0.002	0.256	0.110–0.595	0.002

Data were described as odds ratio (OR) and 95% confidence intervals (CIs). Abbreviation: OA, osteoarthritis; EQ-VAS score, EuroQOL visual analogue scale scores; EQ-5D index, EuroQOL-5dimension index. *p* values were obtained by the composite sample multivariate logistic regression analysis: Model 1, adjusted with age and gender; Model 2, adjusted with age, gender, marriage, education, income, alcohol consumption, and smoking.

## Data Availability

The raw, anonymized data are publicly available from KNHANES 2012 on the official KNHANES web site (https://knhanes.kdca.go.kr/knhanes/sub03/sub03_02_05.do).
